# Device-measured sedentary behaviour and anxiety symptoms during adolescence: a 6-year prospective cohort study

**DOI:** 10.1017/S0033291720004948

**Published:** 2022-10

**Authors:** A. Kandola, G. Lewis, D. P. J. Osborn, B. Stubbs, J. F. Hayes

**Affiliations:** 1 Division of Psychiatry, University College London, London, UK; 2 Camden and Islington NHS Foundation Trust, London, UK; 3 Department of Psychological Medicine, Institute of Psychiatry, Psychology, and Neuroscience, King's College London, London, UK; 4 Physiotherapy Department, South London, and Maudsley National Health Services Foundation Trust, London, UK

**Keywords:** Sedentary behaviour, physical activity, anxiety, depression, adolescence, children, prevention, screen time

## Abstract

**Background:**

Sedentary behaviour is potentially a modifiable risk factor for anxiety disorders, a major source of global disability that typically starts during adolescence. This is the first prospective study of associations between repeated, device-based measures of sedentary behaviour and anxiety symptoms in adolescents.

**Methods:**

A UK cohort with 4257 adolescents aged 12 at baseline (56% female). Main exposures were sedentary behaviour and physical activity measured using accelerometers for 7-days at ages 12, 14, and 16. Primary outcome was anxiety symptom scores at age 18 from a Clinical Interview Schedule-Revised. We used adjusted negative binomial regression and iso-temporal substitution methods to analyse the data.

**Results:**

We found a positive association between sedentary behaviour at ages 12, 14, and 16, with anxiety symptoms at age 18, independent of total physical activity volume. Theoretically replacing an hour of daily sedentary behaviour for light activity at ages 12, 14, and 16, was associated with lower anxiety symptoms by age 18 by 15.9% (95% CI 8.7–22.4), 12.1% (95% CI 3.4–20.1), and 14.7% (95% CI 4–24.2), respectively. Whereas, theoretically replacing an hour of sedentary behaviour with moderate-to-vigorous physical activity was not associated with differences in anxiety symptoms. These results were robust to a series of sensitivity analyses.

**Conclusion:**

Sedentary behaviour is a possible risk factor for increasing anxiety symptoms during adolescence, independent of total physical activity volume. Instead of focusing on moderate-to-vigorous activity, replacing daily sedentary behaviour with light activity during adolescence could be a more suitable method of reducing future anxiety symptoms.

## Introduction

Anxiety is a common mental health problem, characterised by excess worry, fear, and hyperarousal that can be debilitating and interfere with normal daily functioning (Olthuis, Watt, Bailey, Hayden, & Stewart, 2016). Fear is a state of immediate threat and anxiety is the anticipation of future threats (Craske & Stein, 2016). People with anxiety symptoms experience these responses to internal or external triggers out of proportion to the actual danger posed. Anxiety disorders have a widespread global prevalence of around 7.3% in adults (Baxter, Scott, Vos, & Whiteford, 2013) and 6.5% in children and adolescents (Polanczyk, Salum, Sugaya, Caye, & Rohde, 2015). While anxiety disorders frequently co-occur with depression (Kessler et al., 2008), the diagnoses have different symptomologies (Simpson, Neria, Lewis-Fernández, & Schneier, 2010). Collectively, anxiety disorders represent the sixth leading cause of disability worldwide (World Health Organisation, 2017) and are associated with long-term physical health complications, including cardiovascular disease (Batelaan, Seldenrijk, Bot, van Balkom, & Penninx, 2016) and premature mortality (Walker, McGee, & Druss, 2015).

Most anxiety symptoms first occur during adolescence (Kessler, Chiu, Demler, Merikangas, & Walters, 2005) and cause substantial disruptions to the social, education, and family lives of young people (Woodward & Fergusson, 2001) and their functioning in later life (Essau, Lewinsohn, Olaya, & Seeley, 2014). Identifying risk factors for anxiety symptoms that are modifiable during adolescence are essential to strategies for reducing the prevalence and burden of anxiety disorders. Interventions that simultaneously address the long-term physical and mental health outcomes could be particularly important for reducing the mortality gap between people with anxiety disorders and the general population (Firth et al., 2019; Kandola et al., 2018).

Sedentary behaviour refers to any waking activity in a sitting, lying, or reclining position with a low energy expenditure of ⩽1.5 metabolic equivalents, which are standardised units of energy expenditure (Tremblay et al., 2017). Some population-based studies have found that higher levels of self-reported sedentary behaviour and lower levels of physical activity are associated with an increased risk of anxiety symptoms and disorders (Allen, Walter, & Swann, 2019; Hoare, Milton, Foster, & Allender, 2016; McDowell, Dishman, Gordon, & Herring, 2019; Schuch et al., 2019; Teychenne, Costigan, & Parker, 2015). Sedentary behaviour is directly modifiable through increasing physical activity in the day. Several randomised controlled trials have demonstrated that structured physical activity interventions can reduce the symptoms of anxiety in adults with and without anxiety disorders (Gordon, McDowell, Lyons, & Herring, 2017; Herring, O'Connor, & Dishman, 2010; Stonerock, Hoffman, Smith, & Blumenthal, 2015; Stubbs et al., 2017).

However, there has been little research on adolescents. Most studies in this area are cross-sectional and unable to account for reverse causality (Allen et al., 2019; Hoare et al., 2016; Schuch et al., 2019; Teychenne et al., 2015). From four recent systematic reviews (Allen et al., 2019; Hoare et al., 2016; Schuch et al., 2019; Teychenne et al., 2015), only three prospective studies focus on sedentary behaviour (Gunnell et al., 2016; Khouja et al., 2017) or physical activity (Ströhle et al., 2007) and anxiety symptoms in adolescents. These studies used self-reported measures of activity that are subject to mood, attention, and recall biases that reduce the validity of their estimates in adolescents (Adamo, Prince, Tricco, Connor-Gorber, & Tremblay, 2009). Self-reported measures are particularly poor at detecting sedentary behaviour or light-intensity activities, such as walking at a casual pace or light housework (Matthews, Moore, George, Sampson, & Bowles, 2012).

Accelerometers are electromechanical devices that can reliably and continuously estimate activity across a wide range of intensities throughout the day (Ainsworth, Cahalin, Buman, & Ross, 2015). Accelerometer-based studies show that light activity makes up most of waking daily activity, but is progressively displaced by sedentary behaviour throughout adolescence (Colley et al., 2013; Kandola, Lewis, Osborn, Stubbs, & Hayes, 2020; Ortega et al., 2013; Spittaels et al., 2012). We recently found that this activity shift was associated with an increased risk of depressive symptoms by age 18 in the ALSPAC study (Kandola et al., 2020), but we currently lack evidence regarding the impact on anxiety symptoms. Most prospective studies of sedentary behaviour or physical activity interventions focus on depressive symptoms (Bond, Stanton, Wintour, Rosenbaum, & Rebar, 2020; Schuch et al., 2018, 2019), creating a knowledge gap regarding their relationship with anxiety symptoms. Anxiety symptoms are associated with a substantial global health burden and likely have different underlying mechanisms from depressive symptoms that warrant further investigation in their own right.

Physical activity could promote mental health through several mechanisms, such as stimulating neuroplasticity or promoting self-esteem (Kandola, Ashdown-Franks, Hendrikse, Sabiston, & Stubbs, 2019). High sedentary behaviour may forgo some of these possible benefits and cause additional problems that increase mental health risks, such as social isolation or sleep problems (Li et al., 2019; Werneck, Collings, Barboza, Stubbs, & Silva, 2019). While increasing activity will be essential to reducing sedentary behaviour, associations between sedentary behaviour and anxiety symptoms may be independent of total physical activity volume. This independence would suggest that the risks of sedentary behaviour are more than simply a product of low energy expenditure. For example, unengaging sedentary behaviours may induce prolonged bouts of minimal cognitive stimulation that could pose mental health risks, such as watching television (Hallgren et al., 2018; Hallgren, Dunstan, & Owen, 2020).

Advances in traditional regression-based methods are also necessary to account for the reality that reducing sedentary behaviour requires increasing other forms of activity to displace it. Traditional methods only estimate the impact of increasing time in one activity. For example, we estimated in our previous study that a 1 hour increase in daily sedentary behaviour was associated with around a 10% increase in depressive symptoms (Kandola et al., 2020). But this estimation does not account for whether the increase in daily sedentary behaviour comes at the expense of light activity or moderate to vigorous activity, both of which may impact anxiety symptoms differently. Iso-temporal substitution models (ISMs) are a novel method for estimating how substituting time in one activity (e.g. sedentary behaviour) for the time in another (e.g. light activity) affects an outcome (e.g. anxiety symptoms) (Mekary et al., 2013; Mekary, Willett, Hu, & Ding, 2009).

We prospectively investigated associations between sedentary behaviour, light activity, and moderate-to-vigorous physical activity (MVPA) measured using accelerometers during adolescence with anxiety symptoms at age 18 with ISMs. We specifically assessed whether associations between sedentary behaviour and anxiety symptoms were independent of total physical activity volume. To the best of our knowledge, this is the first prospective study to examine associations between device-measured activity and anxiety symptoms in adolescents.

Our directional hypotheses were:

1. Higher sedentary behaviour at ages 12, 14, and 16 is associated with increased anxiety symptoms at age 18
2. Associations between sedentary behaviour and anxiety symptoms are independent of total physical activity volume
3. Substituting periods of daily sedentary behaviour for the light activity or MVPA during adolescence is associated with reductions in anxiety symptoms at age 18

## Methods

### Participants and study design

This is a prospective study with repeated measures using data from the Avon Longitudinal Study of Parents and Children (ALSPAC) cohort. The sample is broadly representative of the general population and full details of the cohort are described elsewhere (Boyd et al., 2013; Fraser et al., 2013). Information about the variables is available through an online data dictionary and variable search tool (http://www.bristol.ac.uk/alspac/researchers/our-data/). The ALSPAC cohort consists of 15 454 women and 14 901 children alive at 12 months of age. All pregnant women in the Avon area of South-West England with an expected delivery date between 1 April 1991 and 31 December 1991, were invited to join the ALSPAC study (Boyd et al., 2013; Fraser et al., 2013). The ALSPAC Law and Ethics committee and the local research ethics committees gave ethical approval for this study. All participants gave informed consent for the use of data collected via questionnaires and clinics following the recommendations of the ALSPAC Ethics and Law Committee at the time. We defined our sample as any participant with complete Clinical Interview Schedule-Revised (CIS-R) anxiety symptoms score at age 17.8 (*n* = 4257), or age 18 for simplicity. Online Supplementary Fig. s1 contains a flowchart of ALSPAC participants for this study.

### Measures

#### Outcome: anxiety symptoms

The primary outcome in our study was anxiety symptoms, measured using a computerised version of the CIS-R anxiety score at age 18. The CIS-R is a common tool for assessing depression and anxiety symptoms in community-based samples, using criteria from the *International Statistical Classification of Diseases, 10^th^ Revision* (ICD-10) (Lewis, Pelosi, Araya, & Dunn, 1992). It performs similarly to diagnosis by a trained psychiatrist and shows good reliability (*r* = 0.90) (Lewis et al., 1992). The anxiety score ranges from 0 to 16 and indicates the number and severity of recent anxiety symptoms, including feelings of anxiety, nervousness, tenseness, or physical symptoms, such as changes in heartrate. We used a discrete-continuous score for our primary outcome as this better reflects how anxiety symptoms manifest than a dichotomised outcome, such as a clinical diagnosis.

To measure baseline anxiety, we used data from the Development and Well-being Assessment (DAWBA) (Goodman, Ford, Richards, Gatward, & Meltzer, 2000), completed by the mother around age 11 (10.8 years) and 14 (14.2 years). The DAWBA consists of questions about mental health symptoms up to the last 6-months following diagnostic criteria from the Diagnostic and Statistical Manual of Mental Disorders, 4th edition (American Psychiatric Association, 1994) and ICD-10. A computer algorithm creates ordered categorical measures to indicate the probability of an anxiety disorder (see Goodman, Heiervang, Collishaw, & Goodman, 2011 for details).

#### Exposure: sedentary behaviour and physical activity

Activity data were collected at ages 11.8, 13.9, and 15.5 (which we refer to as ages 12, 14, and 16) using MTI Actigraph 7164 or 71 256 accelerometers (Actigraph LLC, Fort Walton Beach, FL, USA) worn during waking hours on the right hip for 7 days. We derived total activity volume, time in sedentary behaviour, light activity and MVPA based on counts per minutes (CPM) using methods that we describe in detail elsewhere (Kandola et al., 2020). We used thresholds established by a calibration study in ALSPAC participants (Mattocks et al., 2008) of ⩾3600 CPM for MVPA, 200–3599 CPM for light activity, and ⩽199 CPM for sedentary behaviour. We only included data from participants with at least 10 h of wear time for 3 or more days (Mattocks et al., 2008).

#### Confounding variables

We based our confounding variable adjustments on a Directed Acyclic Graph (DAG), which represents our understanding of the proposed causal associations between sedentary behaviour, physical activity, and anxiety symptoms (online Supplementary Fig. s2). According to this DAG, the necessary adjustments for estimating the total effect of sedentary behaviour on anxiety symptoms include: Sex, ethnicity, social class (maternal manual or non-manual occupation), IQ (measured at age 8), parental psychiatric history (prior diagnosis of depression or schizophrenia), parental education (secondary or degree/higher level education), total physical activity volume (mean daily CPM), baseline anxiety symptoms (DAWBA at age 11 and 14), and total accelerometer wear time. Models at age 16 used the DAWBA at age 14 as the closest baseline measure of anxiety. Total physical activity volume is measured in counts rather than minutes to reduce the risk of collinearity with sedentary behaviour.

Our sensitivity analyses (detailed below) included the covariates: alcohol use and smoking (age 16), baseline depressive symptoms (Short Moods and Feelings Questionnaire at ages 12, 14, and 16), body mass index (BMI) (ages 12, 13, and 16) and physical health status (presence of a severe physical illness before age 17).

#### Analysis

For normally distributed, continuous variables, we calculated means and standard deviations. For non-normally distributed variables, we used medians and interquartile ranges.

For the main analysis, we used negative binomial regression models as the distribution of anxiety scores had a high positive skew and were over-dispersed (online Supplementary Fig. s3). Our main analysis consists of two sets of models. The first set (models 1–9) use single-exposure models (described below) to assess associations between sedentary behaviour, light activity, MVPA, and anxiety symptoms (hypothesis 1) and the possible independence of sedentary behaviour from total physical activity volume (hypothesis 2). The second set (models 10–13) use ISMs to examine substitution effects (hypothesis 3). We present the results of our models as percentage changes in anxiety scores.

We carried out our analysis in Stata 16 (StataCorp LLC).

#### Single-exposure models

Single-exposure models estimate the ‘total’ association between each activity category (sedentary behaviour, light, and MVPA) at ages 12, 14, and 16 and anxiety symptoms at age 18. We used three separate models for each time point of sedentary behaviour measurement. The single-exposure models assess total associations because they do not mutually adjust for other activity categories but do adjust for all other covariates. In addition to crude and adjusted models, we ran additional analyses for sedentary behaviour that also adjusted for total physical activity volume (mean daily CPM). These models assessed whether any association between sedentary behaviour and anxiety symptoms were independent of total physical activity volume (aim 2).

#### Iso-temporal substitution models

The single models assume time is infinite and only estimate the impact of increasing time in one activity on anxiety. These models do not account for the reality that increasing time in one activity must displace time in another activity as time is finite throughout the day.

ISMs estimate the percentage change in anxiety symptoms from substituting a unit of sedentary behaviour time for an equivalent unit of light activity or MVPA time. ISMs use the same set of linear parameters and operate as any generalised linear model. They include all three exposure variables and a total time variable, which is the summation of the three exposure variables. A simple illustration of these parameters using a linear set of parameters would look like:
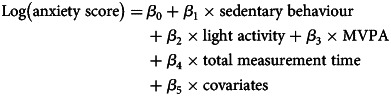


ISMs assume that time in one activity displaces an equal amount of time in another while holding total measurement time (*β*_4_) and other covariates (*β*_5_) constant. As all three exposure variables (*β*_1_, *β*_2_, and *β*_3_) are from the same measure and in the same units of time (e.g. 60-minute blocks), we then drop sedentary behaviour (*β*_1_) from the model. The resulting exposure coefficients (*β*_2_ and *β*_3_) now represent their association with anxiety, absent sedentary behaviour, while total measurement time is still held constant (*β*_4_). We then interpret these coefficients as the consequence of substituting a unit of sedentary behaviour time for a unit of light activity or MVPA time per day on anxiety scores.

We entered activities into these ISMs in units of 60-minutes. As there are no specific guidelines for reducing daily sedentary behaviour, we based this on national recommendations for adolescents to achieve at least 60 min of MVPA per day (Department of Health and Social Care, 2019).

#### Sensitivity analyses and missing data

We conducted a series of sensitivity analyses to explore other possible explanations for our results and test the robustness of our main findings from the ISMs. Some analyses include possible covariates that were measured after the exposure and could represent mediators or colliders, such as physical illness, smoking, and alcohol use. Sensitivity models included: (1) baseline depressive symptoms as a covariate (ages 12, 14, and 16), (2) smoking and alcohol use (age 16 only) as covariates, (3) serious physical illness before age 17, (4) excluding anyone with a possible anxiety disorder at baseline, (5) linear, instead of negative binomial, regression models, (6) sex as an interaction term, (7) baseline BMI as a covariate (ages 12, 13, and 16).

To examine the extent to which any attrition bias from the missing data affected our results (see online Supplementary Fig. s1), we repeated the main analyses in a full cohort with imputed data. The missing data were estimated by multiple imputation models by chained equations (see Supplementary Methods s1 for details). We also calculated the e-value to examine the risk of unmeasured confounding (44). The e-value estimates the minimum strength that an unmeasured confounding variable would have to have to nullify the observed association between exposure and outcome while considering all other measured covariates (VanderWeele & Ding, 2017). The e-value helps to assess the plausibility of unmeasured confounding and contributes towards the evidence for causality (Haneuse, VanderWeele, & Arterburn, 2019).

## Results

### Participants

Our sample included 4257 participants with a complete CIS-R anxiety score at age 18. Over the 6-year follow-up period, the complete case analyses at ages 12, 14, and 16, included 2292, 18,66, and 1128 participants, respectively. Online Supplementary Table s1 contains a comparison of baseline characteristics between included and excluded participants from the full ALSPAC sample.

Table 1 contains participant characteristics and physical activity levels. Overall physical activity levels decline from age 602.33 CPM (s.d. 177.62) to 474.83 CPM (s.d. 158.68) between the ages of 12 and 16. Within the same waking period, sedentary behaviour increases from 7.18 (s.d. 1.10) to 8.72 (s.d. 1.09) hours per day, light activity decreases from 5.43 (s.d. 0.97) to 4.08 (s.d. 0.92) hours per day, and MVPA stays relatively stable.

**Table 1.** Participant characteristics and activity changes of included participants (*n* = 4257)

Characteristic (*n* with complete data)		*n* (%)		
Sex (4257)	Male	1867 (43.86)		
	Female	2390 (56.14)		
Ethnicity (4062)	Caucasian	3889 (95.74)		
	Other	173 (4.26)		
Parental education (4257)	Degree or higher	1207 (28.35)		
	Secondary	3050 (71.65)		
Maternal social class (3626)	Manual	553 (15.25)		
	Non-manual	3073 (84.75)		
Parental psychiatric diagnosis (4257)	Depression or schizophrenia	422 (9.91)		
	No diagnosis	3835 (90.09)		
BMI (3671)	Overweight or obese	270 (7.35)		
	Normal or underweight	3401 (92.65)		
Self-reported serious physical illness before 17 (4130)	Yes	218 (5.27)		
	No	3877 (94.73)		
Alcohol use in last 30 days at age 16 (2922)	1–3 times	2041 (69.85)		
	4–6 times	848 (30.05)		
	7 or 8 times	33 (1.13)		
Smoking status at age 16 (3087)	Never	1694 (54.88)		
	Have tried/former smoker	823 (26.66)		
	Current smoker	570 (18.46)		
Baseline anxiety symptoms (3584)	Possible anxiety (algorithm derived)	65 (1.81)		
	No anxiety	3519 (98.19)		
Baseline depression (3683)	Possible depression (MFQ > = 10)	344 (9.34)		
	No depression (MFQ < 10)	3339 (90.66)		

Physical activity and sedentary behaviour				
Physical activity and sedentary behaviour	Overall physical activity in daily mean CPM (s.d.)	Sedentary behaviour in hours (s.d.)	Light activity in hours (s.d.)	MVPA in minutes (s.d.)
12 (3832)	602.33 (177.62)	7.18 (1.10)	5.43 (0.97)	23.279 (15.37)
14 (2749)	539.12 (181.43)	8.11 (1.14)	4.64 (0.95)	24.273 (16.91)
16 (1504)	474.83 (158.68)	8.72 (1.09)	4.08 (0.92)	23.523 (17.58)

### Single-exposure models

Table 2 shows results from the single-exposure models. The adjusted models without total activity volume (models 2, 5, and 8) suggest that an additional 60-minutes of sedentary behaviour at ages 12, 14, and 16, was associated with an 18.22% (95% CI 10.10–26.87), 10.19% (95% CI 1.37–19.79), and 15.75% (95% CI 3.71–29.12) higher anxiety score at age 18. An additional 60-minutes of light activity at 12, 14, and 16, was associated with a −16.82% (95% CI −22.99 to −10.16), −11.57% (95% CI −19.30 to −2.98), and −14.81% (95% CI −24.29 to −4.14) decrease in anxiety score. The model estimates suggest no association between MVPA and anxiety scores in this sample.

**Table 2.** Associations between sedentary behaviour, light activity, and MVPA and anxiety symptoms in single negative binomial models

Age	Model #	Model (*n*)	CIS-R anxiety symptoms score								
			Per 60 min of daily sedentary behaviour			Per 60 min of daily light activity			Per 60 min of daily MVPA		
			% change	*p*	95% CI	%Δ	*p*	95% CI	%Δ	*p*	95% CI
12	1	Unadjusted (2292)	20	<0.001	13.09–27.88	−18.2	<0.001	−23.5 to −12.46	−46.6	<0.001	−59.40 to −29.79
	2	Adjusted without total activity (2292)	18.22	<0.001	10.10–26.87	−16.82	<0.001	−22.99 to −10.16	−22.11	0.109	−42.5 to 5.67
	3	Adjusted with total activity (2292)	21.12	0.001	8.36–35.56	–	–	–	–	–	–
14	4	Unadjusted (1866)	13.20	<0.001	6.02–20.87	−14.31	<0.001	−20.94 to −7.12	16.82	0.187	−36.64 to 9.21
	5	Adjusted without total activity (1866)	10.19	0.023	1.37–19.79	−11.57	0.010	−19.30 to −2.98	2.26	0.881	−23.7 to 36.88
	6	Adjusted with total activity	16.19	0.019	2.47–31.74	–	–	–	–	–	–
16	7	Unadjusted (1128)	15.99	0.001	6.34–26.51	−16.06	0.001	−24.20 to −6.92	−16.63	0.224	−37.71 to 11.66
	8	Adjusted without total activity (1128)	15.75	0.009	3.71–29.12	−14.81	0.008	−24.29 to −4.14	−7.62	0.646	−33.83 to 29.05
	9	Adjusted with total activity	17.43	0.041	0.67–36.99	–	–	–	–	–	–

% change: Percentage change in anxiety score based on incident rate ratios; 95% CI: 95% confidence intervals; MVPA: moderate-to-vigorous physical activity.

Adjusted without total activity (models 2, 5, and 8): Conditioned on sex, ethnicity, maternal social class, baseline anxiety, parental psychiatric history, parental education, IQ, and total wear time.

Adjusted with total activity (models 3, 6, and 9): Conditioned on same as models 2, 5, and 8, with total activity volume (average daily CPM).

When adjusting for total activity (models 3, 6, and 9), sedentary behaviour at ages 12, 14, and 16 was associated with a 21.12% (95% CI 8.36–35.65), 16.19% (95% CI 2.47–31.74), and a 17.43% (95% CI 0.67–36.99) higher anxiety scores at age 18.

### Iso-temporal substitution models

Table 3 displays results from the ISMs. These models suggest that substituting 60 min of sedentary behaviour for 60 min of light activity at ages 12, 14, and 16, was associated with a 16.4% (95% CI 9.4–22.7), 12.1% (95% CI 3.4–20.1), and 14.7% (95% CI 4–24.2) reduction in anxiety scores at 18. We found no associations between MVPA and anxiety.

**Table 3.** Iso-temporal substitution models for replacing sedentary behaviour with light activity and MVPA

Age	Model #	Substitute 60 min of ↓	with 60 min of →	Activity category			
				Light activity		MVPA	
		Activity category	% change	95% CI	%Δ	95% CI
12	10	Sedentary		−16.41	−22.79 to −9.49	−7.62	−32.42 to 26.38
14	11			−12.17	−20.11 to −3.46	−10.99	−17.71 to 49.53
16	12			−14.74	−24.24 to −4.05	−5.91	−32.52 to 31.47

% change: Percentage change in anxiety score based on incident rate ratios 95% CI: 95% confidence intervals; MVPA: moderate-to-vigorous physical activity.

Adjustment for all models: light activity, MVPA, total measurement time (SB + light activity + MVPA), sex, ethnicity, maternal social class, anxiety disorder (ages 10 and 14), parental psychiatric history, parental education, and IQ.

### Sensitivity analyses

We reran our main analyses in a full cohort (*n* = 4257) with imputed missing data (Methods s1) and the results did not substantially differ from our main findings (online Supplementary Table s2). There were also no substantial differences in the results of all other sensitivity analyses and our main findings, and we found no evidence of an interaction with sex. Sensitivity analyses results are presented in online Supplementary Tables s2–s8.

The e-values (rate ratios) for the point estimate and lower confidence bound were 1.68 and 1.44 at age 12, 1.53 and 1.23 at age 14, and 1.62 and 1.25 at age 16, respectively. To nullify the observed association, an unmeasured confounding variable must be associated with both the exposure and outcome by a risk ratio of at least the e-value of each time point, having conditioned on the other confounding variables. For example, an unmeasured confounding variable would need to be associated with sedentary behaviour and anxiety score by a risk ratio of at least 1.68 at age 12, independent of the other confounding variables in the model. Most risk ratios for included covariates range between 1 and 1.3 at age 12, except for sex (IRR = 1.59, 95% CI 1.37–1.84).

## Discussion

### Main findings

This population-based study is the first to prospectively examine associations between device-measured sedentary behaviour and anxiety symptoms in adolescents. We consistently found that higher sedentary behaviour at ages 12, 14, and 16 was associated with higher anxiety symptoms at age 18. Higher light activity during the same period was associated with a decrease in anxiety symptoms. After adjusting for physical activity, an hour of daily sedentary behaviour during adolescence was independently associated with 16–21% higher in anxiety symptoms at age 18. Our use of ISM techniques demonstrates that theoretically substituting an hour of daily sedentary behaviour for light activity during adolescence was associated with a 12–16% reduction in anxiety symptoms at age 18. We found no associations between MVPA and anxiety. There were no substantial changes to these results following a series of sensitivity analyses or in the cohort following multiple imputations for missing data.

These results support previous cross-sectional and prospective findings that self-reported high sedentary behaviour is associated with a greater risk of anxiety symptoms in adolescents (Allen et al., 2019; Hoare et al., 2016; Schuch et al., 2019; Teychenne et al., 2015). Sedentary behaviour is an established risk factor for physical health (Biswas et al., 2015) and our findings suggest it may also be a risk factor for anxiety symptoms independently of physical activity. Activity will be essential for reducing sedentary behaviour, but these findings suggest that other factors than energy expenditure are relevant to its association with anxiety symptoms. We previously found that sedentary behaviour is a possible risk factor for depressive symptoms in adolescents, where we did not assess sedentary behaviour as independent from physical activity (Kandola et al., 2020). Here we showed that the associations between sedentary behaviour and anxiety symptoms are independent of baseline depressive symptoms.

For example, substituting mentally-passive (e.g. watching television) for mentally-active (e.g. reading) sedentary behaviours is associated with a reduced risk of depression in adults (Hallgren et al., 2020). Engaging activities could help to distract young people from pathological thought patterns leading to states of anxiety. Stimulating activities could also approximate a form of cognitive training that elicits some resilience to attentional biases associated with developing anxiety symptoms in young people, such as threat detection (Telzer et al., 2008; Waters, Henry, Mogg, Bradley, & Pine, 2010).

The timing and bouts of activity may also be relevant. For example, long bouts of sitting could increase the duration within which pathological thought patterns might occur and develop into anxiety. Recent studies have found that breaking up prolonged bouts of sitting using light activity in adults have benefits for the brain and mental health, such as reducing fatigue and promoting brain plasticity and cognitive performance (Bojsen-Møller, Ekblom, Tarassova, Dunstan, & Ekblom, 2020; Wennberg et al., 2016; Wheeler et al., 2020). These benefits may accumulate to reduce the risk of anxiety symptoms developing. Young people who frequently break up bouts of sitting with activity could reduce this risk while still having a similar overall activity level. It may also interrupt other behaviours that occur with long bouts of sitting, which might also increase the risk of anxiety symptoms. For example, watching television is associated with a less healthy diet, including higher consumption of energy-dense snacks and sugar-sweetened drinks (Hobbs, Pearson, Foster, & Biddle, 2015), which may increase the risk of anxiety symptoms in adolescents (Oddy et al., 2018).

While the biological mechanisms underlying associations between sedentary behaviour and anxiety symptoms will overlap with physical activity, there may also be some unique pathways. For example, avoiding long bouts of sedentary behaviour could maintain constant mitochondrial activity throughout the day, reducing the risk of mitochondrial dysfunction in the brain leading to anxiety symptoms (Filiou & Sandi, 2019). It is worth noting that research to identify possible biological mechanisms that differentiate the influence of sedentary behaviour from physical activity on health is an emerging area (Thyfault, Du, Kraus, Levine, & Booth, 2015).

### Strengths and limitations

There are several strengths to this study, including the use of accelerometers, a prospective, repeated measures study design with a 6-year follow up, and large sample size. Strengths in our analysis include using ISMs to account for the reciprocal relationship between changes in time-use variables. These methods produce a more realistic estimation of how interventions to reduce sedentary behaviour using different intensities of activity might affect anxiety symptoms. We also included adjustments for baseline anxiety symptoms to lower the risk of reverse causation and adequate adjustment of total physical activity volume. We used several sensitivity analyses, including calculating e-values to assess the strength of unmeasured confounding necessary to nullify our results, baseline depressive symptoms to examine anxiety symptoms independently, and multiple imputation models to account for potential selection bias due to attrition. The use of DAGs determined *a priori* also improve our ability to assess causal associations in the data.

A limitation of our study is the lack of 24-hour activity, which means that our ISMs only account for the waking portion of the day. Participants did not wear accelerometers while sleeping, so we were unable to examine how substituting sedentary behaviour for sleep might affect the risk of anxiety symptoms. There could also be gaps in the data if participants did not wear their devices for all waking behaviours. There is a further risk that our results are confounded by other unmeasured factors, such as social support, self-esteem, or physical health at baseline. Baseline adjustment was for the possible incidence of an anxiety disorder, which overlooks participants with sub-threshold anxiety symptoms. Baseline assessments of anxiety were also completed by the mother, rather than the participant. There was no assessment of anxiety symptoms at age 16, so in the age 16 models, we used anxiety symptom scores from age 14, which is a further limitation.

To assess anxiety symptoms independently of depressive symptoms, we adjusted for baseline depressive symptoms in our sensitivity analyses. However, there may still have been an overlap in the outcome of depressive and anxiety symptoms. The magnitude of the e-value relative to included confounding variables here suggests that an unmeasured confounding variable is unlikely to nullify the observed association between sedentary behaviour and anxiety symptoms. However, multiple unmeasured confounding variables may accumulate to impact our findings.

There was also substantial attrition within our sample during the study period that could have caused selection bias. Results did not differ in our multiple imputation models, which does not indicate a high risk of attrition bias in our sample. However, we only imputed missing data from the subsample with completed CIS-R anxiety scores at age 18 (*n* = 4257), not the entire ALSPAC sample (*n* = 14 901). Differences between our sample and the larger ALSPAC sample could have influenced our results. Participants also had low MVPA levels, with the average being around 40 min lower than the nationally recommended guidelines of 60 min of daily MVPA for adolescents in the UK. This may have contributed to the lack of an association between replacing sedentary behaviour with MVPA and changes in anxiety symptoms.

Accelerometers provide reliable estimates of activity, but they cannot record posture and differentiate between sitting and standing. Standing behaviours may be misclassified as sedentary behaviour in our study. Thigh-worn devices are preferable for recording sedentary behaviour, such as ActivPAL (PAL Technologies Ltd., Glasgow, UK). However, misclassifying light activity (standing) would increase sedentary behaviour, and its true association with anxiety symptoms could be even larger.

A broader issue with most devices is their inability to record the type and context of activities. This limitation prevented us from investigating how different types of sedentary behaviour are associated with anxiety, such as mentally-active *v.* mentally-passive behaviours (Hallgren et al., 2020). However, the use of accelerometers still represents a methodological improvement in self-report measures for quantifying aspects of activity, such as time in sedentary behaviour (Atkin et al., 2012).

### Future directions and conclusion

Our findings suggest that sedentary behaviour could be a risk factor for anxiety symptoms in adolescents that is modifiable through light activity. These findings produce new insights to relate specifically to the development of anxiety disorders. Most research focuses only on depression, despite anxiety being a major cause of global disability (World Health Organisation, 2017). Sedentary behaviour may be a risk factor for both depressive and anxiety symptoms. Future research focusing on both paradigms together as common mental disorders may highlight common pathways. However, our results suggest that there may be independent pathways linking sedentary behaviour to anxiety symptoms, and the paucity of direct research into anxiety symptoms warrants further investigation.

There is a need to evaluate public health strategies and interventions to reduce anxiety symptoms in adolescents using light activity to replace sedentary behaviour. While just 29% of adolescents achieve national MVPA guidelines in developed nations (Steene-Johannessen et al., 2020), efforts to increase light activity could be more successful. Compared with MVPA, light activity is less effortful and more pleasurable for most people, which is likely to stimulate higher motivation (Ekkekakis, Parfitt, & Petruzzello, 2011). Light activity is also sustainable over extended periods and does not require designated time during the day. Simple changes to incorporate more light activity at school could include standing desks or active breaks during classes. Approaches at home could include standing up during commercial breaks or doing house chores while watching television, more frequent trips to pick up groceries, or walking during phone calls.

Strong public health messaging on the importance of light activity should be made in tandem with the MVPA guidelines, which have essential physical health and developmental implications. Simply increasing movement will likely benefit more young people. It should be possible to increase light activity beyond the current target of 1 hour for MVPA, which may have a substantial impact on anxiety. For example, our models suggest that substituting 2 hours of sedentary behaviour for light activity per day during adolescence could lead to a 24–32% reduction in anxiety symptoms by age 18.

We also found that associations between sedentary behaviour and anxiety symptoms were independent of activity. Some sedentary behaviours may be particularly detrimental and represent specific targets for intervention, such as watching television. More research is necessary to assess the differential impact of various sedentary behaviours on anxiety symptoms.

## Data Availability

Details for accessing the data used in this study can be found on the ALSPAC website: http://www.bristol.ac.uk/alspac/researchers/our-data/.

## Supplementary material

For supplementary material accompanying this paper visit https://doi.org/10.1017/S0033291720004948. click here to view supplementary material

## References

[ref1] Adamo, K. B., Prince, S. A., Tricco, A. C., Connor-Gorber, S., & Tremblay, M. (2009). A comparison of indirect versus direct measures for assessing physical activity in the pediatric population: A systematic review. International Journal of Pediatric Obesity, 4(1), 2–27. doi:10.1080/17477160802315010. 18720173

[ref2] Ainsworth, B., Cahalin, L., Buman, M., & Ross, R. (2015). The current state of physical activity assessment tools. Progress in Cardiovascular Diseases, 57(4), 387–395. doi:10.1016/J.PCAD.2014.10.005. 25446555

[ref3] Allen, M. S., Walter, E. E., & Swann, C. (2019). Sedentary behaviour and risk of anxiety: A systematic review and meta-analysis. Journal of Affective Disorders, 242, 5–13. doi:10.1016/J.JAD.2018.08.081. 30170238

[ref4] American Psychiatric Association (1994). Diagnostic and statistical manual of mental disorders (4th ed.). American Psychiatric Association: Washington, D.C.

[ref5] Atkin, A. J., Gorely, T., Clemes, S. A., Yates, T., Edwardson, C., Brage, S., … Biddle, S. J. (2012). Methods of measurement in epidemiology: Sedentary behaviour. International Journal of Epidemiology, 41(5), 1460–1471. doi:10.1093/ije/dys118. 23045206 PMC3465769

[ref6] Batelaan, N. M., Seldenrijk, A., Bot, M., van Balkom, A. J. L. M., & Penninx, B. W. J. H. (2016). Anxiety and new onset of cardiovascular disease: Critical review and meta-analysis. British Journal of Psychiatry, 208(03), 223–231. doi:10.1192/bjp.bp.114.156554. 26932485

[ref7] Baxter, A. J., Scott, K. M., Vos, T., & Whiteford, H. A. (2013). Global prevalence of anxiety disorders: A systematic review and meta-regression. Psychological Medicine, 43(5), 897–910. doi: 10.1017/S003329171200147X. 22781489

[ref8] Biswas, A., Oh, P. I., Faulkner, G. E., Bajaj, R. R., Silver, M. A., Mitchell, M. S., & Alter, D. A. (2015). Sedentary time and its association with risk for disease incidence, mortality, and hospitalization in adults. Annals of Internal Medicine, 162(2), 123. doi:10.7326/M14-1651. 25599350

[ref9] Bojsen-Møller, E., Ekblom, M. M., Tarassova, O., Dunstan, D. W., & Ekblom, O. (2020). The effect of breaking up prolonged sitting on paired associative stimulation-induced plasticity. Experimental Brain Research, 238(11), 2497–2506. doi:10.1007/s00221-020-05866-z. 32860117 PMC7541377

[ref10] Bond, G., Stanton, R., Wintour, S. A., Rosenbaum, S., & Rebar, A. L. (2020). Do exercise trials for adults with depression account for comorbid anxiety? A systematic review. Mental Health and Physical Activity, 18, 100320. doi:10.1016/j.mhpa.2020.100320.

[ref11] Boyd, A., Golding, J., Macleod, J., Lawlor, D. A., Fraser, A., Henderson, J., … Smith, G. D. (2013). Cohort profile: The ‘Children of the 90s’-the index offspring of the Avon Longitudinal Study of Parents and Children. doi:10.1093/ije/dys064. PMC3600618 22507743

[ref12] Colley, R. C., Garriguet, D., Janssen, I., Wong, S. L., Saunders, T. J., Carson, V., & Tremblay, M. S. (2013). The association between accelerometer-measured patterns of sedentary time and health risk in children and youth: Results from the Canadian Health Measures Survey. BMC Public Health, 13(1), 200. doi: 10.1186/1471-2458-13-200. 23497190 PMC3599834

[ref13] Craske, M. G., & Stein, M. B.. (2016). Anxiety. Lancet, 388(10063), 3048–3059. doi:10.1016/S0140-6736(16)30381-6. 27349358

[ref14] Department of Health and Social Care (2019). UK Chief medical officers’ physical activity guidelines. Government, UK: Department of Health and Social Care. Retrieved from https://assets.publishing.service.gov.uk/government/uploads/system/uploads/attachment_data/file/832868/uk-chief-medical-officers-physical-activity-guidelines.pdf.

[ref15] Ekkekakis, P., Parfitt, G., & Petruzzello, S. J. (2011). The pleasure and displeasure people feel when they exercise at different intensities: Decennial update and progress towards a tripartite rationale for exercise intensity prescription. Sports Medicine. Springer, 41(8), 641–671. doi:10.2165/11590680-000000000-00000. 21780850

[ref16] Essau, C. A., Lewinsohn, P. M., Olaya, B., & Seeley, J. R. (2014). Anxiety disorders in adolescents and psychosocial outcomes at age 30. Journal of Affective Disorders, 163, 125–132. doi: 10.1016/j.jad.2013.12.033. 24456837 PMC4028371

[ref17] Filiou, M. D., & Sandi, C. (2019). Anxiety and brain mitochondria: A bidirectional crosstalk. Trends in Neurosciences, 42(9), 573–588. doi:10.1016/j.tins.2019.07.002. 31362874

[ref18] Firth, J., Siddiqi, N., Koyanagi, A., Siskind, D., Rosenbaum, S., Galletly, C., … Stubbs, B. (2019). The Lancet Psychiatry Commission: A blueprint for protecting physical health in people with mental illness. The Lancet. Psychiatry, 6(8), 675–712. doi:10.1016/S2215-0366(19)30132-4. 31324560

[ref19] Fraser, A., Macdonald-Wallis, C., Tilling, K., Boyd, A., Golding, J., Davey Smith, G., … Lawlor, D. A. (2013). Cohort profile: The Avon Longitudinal Study of Parents and Children: ALSPAC mothers cohort. International Journal of Epidemiology, 42(1), 97–110. doi:10.1093/ije/dys066. 22507742 PMC3600619

[ref20] Goodman, R., Ford, T., Richards, H., Gatward, R., & Meltzer, H. (2000). The Development and Well-Being Assessment: Description and initial validation of an integrated assessement of child and adolescent psychopathology. Journal of Child Psychology and Psychiatry and Allied Disciplines, 41(5), 645–655. doi:10.1017/S0021963099005909. 10946756

[ref21] Goodman, A., Heiervang, E., Collishaw, S., & Goodman, R. (2011). The “DAWBA bands” as an ordered-categorical measure of child mental health: Description and validation in British and Norwegian samples. Social Psychiatry and Psychiatric Epidemiology, 46(6), 521–532. doi:10.1007/s00127-010-0219-x. 20376427

[ref22] Gordon, B. R., McDowell, C. P., Lyons, M., & Herring, M. P. (2017). The effects of resistance exercise training on anxiety: A meta-analysis and meta-regression analysis of randomized controlled trials. Sports Medicine, 47(12), 2521–2532. doi:10.1007/s40279-017-0769-0. 28819746

[ref23] Gunnell, K. E., Flament, M. F., Buchholz, A., Henderson, K. A., Obeid, N., Schubert, N., & Goldfield, G. S. (2016). Examining the bidirectional relationship between physical activity, screen time, and symptoms of anxiety and depression over time during adolescence. Preventive Medicine, 88, 147–152. doi:10.1016/j.ypmed.2016.04.002. 27090920

[ref24] Hallgren, M., Dunstan, D. W., & Owen, N. (2020). Passive versus mentally active sedentary behaviors and depression. Exercise and Sport Sciences Reviews, 48(1), 20–27. doi:10.1249/JES.0000000000000211. 31663866

[ref25] Hallgren, M., Nguyen, T.-T.-D., Owen, N., Stubbs, B., Vancampfort, D., Lundin, A., … Lagerros, Y. T. (2020). Cross-sectional and prospective relationships of passive and mentally active sedentary behaviours and physical activity with depression. The British Journal of Psychiatry, 217(2), 413–419. doi:10.1192/bjp.2019.60. 30895922

[ref26] Hallgren, M., Owen, N., Stubbs, B., Zeebari, Z., Vancampfort, D., Schuch, F., … Trolle Lagerros, Y. (2018). Passive and mentally-active sedentary behaviors and incident major depressive disorder: A 13-year cohort study. Journal of Affective Disorders, 241, 579–585. doi:10.1016/J.JAD.2018.08.020. 30170310

[ref27] Haneuse, S., VanderWeele, T. J., & Arterburn, D. (2019). Using the E-value to assess the potential effect of unmeasured confounding in observational studies. JAMA, 321(6), 602–603. doi:10.1001/jama.2018.21554. 30676631

[ref28] Herring, M. P., O'Connor, P. J., & Dishman, R. K. (2010). The effect of exercise training on anxiety symptoms among patients. Archives of Internal Medicine, 170(4), 321. doi:10.1001/archinternmed.2009.530. 20177034

[ref29] Hoare, E., Milton, K., Foster, C., & Allender, S. (2016). The associations between sedentary behaviour and mental health among adolescents: A systematic review. International Journal of Behavioral Nutrition and Physical Activity, 13(1), 108. doi:10.1186/s12966-016-0432-4. 27717387 PMC5055671

[ref30] Hobbs, M., Pearson, N., Foster, P. J., & Biddle, S. J. H. (2015). Sedentary behaviour and diet across the lifespan: An updated systematic review. British Journal of Sports Medicine, 49(18), 1179 LP–1188. doi:10.1136/bjsports-2014-093754. 25351783

[ref31] Kandola, A., Ashdown-Franks, G., Hendrikse, J., Sabiston, C. M., & Stubbs, B. (2019). Physical activity and depression: Towards understanding the antidepressant mechanisms of physical activity. Neuroscience & Biobehavioral Reviews, 107, 525–539. doi:10.1016/J.NEUBIOREV.2019.09.040. 31586447

[ref32] Kandola, A., Lewis, G., Osborn, D. P. J., Stubbs, B., & Hayes, J. F. (2020). Depressive symptoms and objectively measured physical activity and sedentary behaviour throughout adolescence: A prospective cohort study. The Lancet. Psychiatry, 7, 262–271. 32059797 10.1016/S2215-0366(20)30034-1 PMC7033559

[ref33] Kandola, A., Vancampfort, D., Herring, M., Rebar, A., Hallgren, M., Firth, J., … Stubbs, B. (2018). Moving to beat anxiety: Epidemiology and therapeutic issues with physical activity for anxiety. Current Psychiatry Reports, 20(8), 63. doi:10.1007/s11920-018-0923-x. 30043270 PMC6061211

[ref34] Kessler, R. C., Chiu, W. T., Demler, O., Merikangas, K. R., & Walters, E. E. (2005). Prevalence, severity, and comorbidity of 12-month DSM-IV disorders in the National Comorbidity Survey Replication. Archives of General Psychiatry, 62(6), 617–627. doi:10.1001/archpsyc.62.6.617. 15939839 PMC2847357

[ref35] Kessler, R. C., Gruber, M., Hettema, J. M., Hwang, I., Sampson, N., & Yonkers, K. A. (2008). Co-morbid major depression and generalized anxiety disorders in the National Comorbidity Survey follow-up. Psychological Medicine, 38(03), 365–374. doi:10.1017/S0033291707002012. 18047766 PMC2745899

[ref36] Khouja, J., Munafò, M., Tilling, K., Wiles, N., Joinson, C., Etchells, P., … Cornish, R. (2017). Is increased screen time associated with the development of anxiety or depression in young people? European Psychiatry, 41, S530–S531. doi:10.1016/j.eurpsy.2017.01.719. PMC6337855 30654771

[ref37] Lewis, G., Pelosi, A. J., Araya, R., & Dunn, G. (1992). Measuring psychiatric disorder in the community: A standardized assessment for use by lay interviewers. Psychological Medicine, 22(2), 465–486. doi: 10.1017/s0033291700030415. 1615114

[ref38] Li, X., Buxton, O. M., Lee, S., Chang, A. M., Berger, L. M., & Hale, L. (2019). Sleep mediates the association between adolescent screen time and depressive symptoms. Sleep Medicine, 57, 51–60. doi:10.1016/j.sleep.2019.01.029. 30897456 PMC6511486

[ref39] Matthews, C. E., Moore, S. C., George, S. M., Sampson, J., & Bowles, H. R. (2012). Improving self-reports of active and sedentary behaviors in large epidemiologic studies. Exercise and Sport Sciences Reviews, 40(3), 1. doi:10.1097/JES.0b013e31825b34a0. PMC3388604 22653275

[ref40] Mattocks, C., Ness, A. R., Leary, S. D., Tilling, K., Blair, S. N., Shield, & J., … Riddoch, (2008). Use of accelerometers in a large field-based study of children: Protocols, design issues, and effects on precision. Journal of Physical Activity and Health, 5(Suppl 1), S98–S111. doi: 10.1123/jpah.5.s1.s98. 18364528

[ref41] McDowell, C. P., Dishman, R. K., Gordon, B. R., & Herring, M. P. (2019). Physical activity and anxiety: A systematic review and meta-analysis of prospective cohort studies. American Journal of Preventive Medicine, 57(4), 545–556. doi:10.1016/J.AMEPRE.2019.05.012. 31542132

[ref42] Mekary, R. A., Lucas, M., Pan, A., Okereke, O. I., Willett, W. C., Hu, F. B., & Ding, E. L. (2013). Isotemporal substitution analysis for physical activity, television watching, and risk of depression. American Journal of Epidemiology, 178(3), 474–483. doi:10.1093/aje/kws590. 23785112 PMC3727339

[ref43] Mekary, R. A., Willett, W. C., Hu, F. B., & Ding, E. L. (2009). Isotemporal substitution paradigm for physical activity epidemiology and weight change. American Journal of Epidemiology, 170(4), 519–527. doi:10.1093/aje/kwp163. 19584129 PMC2733862

[ref44] Oddy, W. H., Allen, K. L., Trapp, G. S. A., Ambrosini, G. L., Black, L. J., Huang, R.-C., … Mori, T. A. (2018). Dietary patterns, body mass index and inflammation: Pathways to depression and mental health problems in adolescents. Brain. Behavior and Immunity, 69, 428–439. doi:10.1016/J.BBI.2018.01.002. 29339318

[ref45] Olthuis, J. V., Watt, M. C., Bailey, K., Hayden, J. A., & Stewart, S. H. (2016). Therapist-supported internet cognitive behavioural therapy for anxiety disorders in adults. Cochrane Database of Systematic Reviews, 3, CD011565. doi:10.1002/14651858.CD011565.pub2. 26968204 PMC7077612

[ref46] Ortega, F. B., Konstabel, K., Pasquali, E., Ruiz, J. R., Hurtig-Wennlöf, A., Mäestu, J., … Sjöström, M. (2013). Objectively measured physical activity and sedentary time during childhood, adolescence and young adulthood: A cohort study. PLoS One, 8(4), e60871. doi: 10.1371/journal.pone.0060871. 23637772 PMC3634054

[ref47] Polanczyk, G. V., Salum, G. A., Sugaya, L. S., Caye, A., & Rohde, L. A. (2015). Annual research review: A meta-analysis of the worldwide prevalence of mental disorders in children and adolescents. Journal of Child Psychology and Psychiatry and Allied Disciplines, 56(3), 345–365. doi:10.1111/jcpp.12381. 25649325

[ref48] Schuch, F. B., Stubbs, B., Meyer, J., Heissel, A., Zech, P., Vancampfort, D., … Hiles, S. A. (2019). Physical activity protects from incident anxiety: A meta-analysis of prospective cohort studies. Depression and Anxiety, 36(9), 846–858. doi:10.1002/da.22915. 31209958

[ref49] Schuch, F.B., Vancampfort, D., Firth, J., Rosenbaum, S., Ward, P.B., Silva, E., … … Stubbs, B. (2018). Physical activity and incident depression: A meta-analysis of prospective cohort studies. American Journal of Psychiatry, 175(7), 631–648. doi:10.1176/appi.ajp.2018.17111194. 29690792

[ref50] Simpson, H. B., Neria, Y., Lewis-Fernández, R., & Schneier, F. (2010). Anxiety disorders: Theory, research, and clinical perspectives. In H. B. Simpson, Y. Neria, R. Lewis-Fernandez, & F. Schneier (Eds.), Anxiety disorders: Theory, research, and clinical perspectives (pp. 1–394). Cambridge: Cambridge University Press. doi:10.1017/CBO9780511777578.

[ref51] Spittaels, H., Van Cauwenberghe, E., Verbestel, V., De Meester, F., Van Dyck, D., Verloigne, M., … De Bourdeaudhuij, I. (2012). Objectively measured sedentary time and physical activity time across the lifespan: A cross-sectional study in four age groups. International Journal of Behavioral Nutrition and Physical Activity, 9(1), 149. doi:10.1186/1479-5868-9-149. 23249449 PMC3542099

[ref52] Steene-Johannessen, J., Hansen, B. H., Dalene, K. E., Kolle, E., Northstone, K., Møller, N. C., … Ekelund, U. (2020). Variations in accelerometry measured physical activity and sedentary time across Europe – harmonized analyses of 47497 children and adolescents. International Journal of Behavioral Nutrition and Physical Activity, 17(1), 38. doi:10.1186/s12966-020-00930-x. 32183834 PMC7079516

[ref53] Stonerock, G. L., Hoffman, B. M., Smith, P. J., & Blumenthal, J. A. (2015). Exercise as treatment for anxiety: Systematic review and analysis. Annals of Behavioral Medicine: A Publication of the Society of Behavioral Medicine, 49(4), 542–556. doi:10.1007/s12160-014-9685-9. 25697132 PMC4498975

[ref54] Ströhle, A., Höfler, M., Pfister, H., Müller, A. G., Hoyer, J., Wittchen, H. U., & Lieb, R. (2007). Physical activity and prevalence and incidence of mental disorders in adolescents and young adults. Psychological Medicine, 37(11), 1657–1666. doi:10.1017/S003329170700089X. 17579930

[ref55] Stubbs, B., Vancampfort, D., Rosenbaum, S., Firth, J., Cosco, T., Veronese, N., … Schuch, F. B. (2017). An examination of the anxiolytic effects of exercise for people with anxiety and stress-related disorders: A meta-analysis. Psychiatry Research, 249, 102–108. doi:10.1016/j.psychres.2016.12.020. 28088704

[ref56] Telzer, E. H., Mogg, K., Bradley, B. P., Mai, X., Ernst, M., Pine, D. S., & Monk, C. S. (2008). Relationship between trait anxiety, prefrontal cortex, and attention bias to angry faces in children and adolescents. Biological Psychology, 79(2), 216–222. doi:10.1016/J.BIOPSYCHO.2008.05.004. 18599179 PMC2574721

[ref57] Teychenne, M., Costigan, S. A., & Parker, K. (2015). The association between sedentary behaviour and risk of anxiety: A systematic review. BMC Public Health, 15(1), 513. doi:10.1186/s12889-015-1843-x. 26088005 PMC4474345

[ref58] Thyfault, J. P., Du, M., Kraus, W. E., Levine, J. A., & Booth, F. W.. (2015). Physiology of sedentary behavior and its relationship to health outcomes. Medicine and science in sports and exercise, 47(6), 1301–1305. 10.1249/MSS.0000000000000518. 25222820 PMC4362885

[ref59] Tremblay, M. S., Aubert, S., Barnes, J. D., Saunders, T. J., Carson, V., Latimer-Cheung, A. E., … Chinapaw, M. J. M. (2017). Sedentary behavior research network (SBRN) – terminology consensus project process and outcome. International Journal of Behavioral Nutrition and Physical Activity, 14(1), 75. doi: 10.1186/s12966-017-0525-8. 28599680 PMC5466781

[ref60] VanderWeele, T. J., & Ding, P. (2017). Sensitivity analysis in observational research: Introducing the E-value. Annals of Internal Medicine, 167(4), 268–274. doi:10.7326/M16-2607. 28693043

[ref61] Walker, E. R., McGee, R. E., & Druss, B. G. (2015). Mortality in mental disorders and global disease burden implications. JAMA Psychiatry, 72(4), 334. doi:10.1001/jamapsychiatry.2014.2502. 25671328 PMC4461039

[ref62] Waters, A. M., Henry, J., Mogg, K., Bradley, B. P., & Pine, D. S. (2010). Attentional bias towards angry faces in childhood anxiety disorders. Journal of Behavior Therapy and Experimental Psychiatry, 41(2), 158–164. doi:10.1016/J.JBTEP.2009.12.001. 20060097

[ref63] Wennberg, P., Boraxbekk, C. J., Wheeler, M., Howard, B., Dempsey, P. C., Lambert, G., … Dunstan, D. W. (2016). Acute effects of breaking up prolonged sitting on fatigue and cognition: A pilot study. BMJ Open, 6(2), e009630. doi:10.1136/bmjopen-2015-009630. PMC4769400 26920441

[ref64] Werneck, A. O., Collings, P. J., Barboza, L. L., Stubbs, B., & Silva, D. R. (2019). Associations of sedentary behaviors and physical activity with social isolation in 100839 school students: The Brazilian Scholar Health Survey. General Hospital Psychiatry, 59, 7–13. doi:10.1016/j.genhosppsych.2019.04.010. 31054464

[ref65] Wheeler, M. J., Green, D. J., Ellis, K. A., Cerin, E., Heinonen, I., Naylor, L. H., … Dunstan, D. W. (2020). Distinct effects of acute exercise and breaks in sitting on working memory and executive function in older adults: A three-arm, randomised cross-over trial to evaluate the effects of exercise with and without breaks in sitting on cognition. British Journal of Sports Medicine, 54(13), 776–781. doi:10.1136/bjsports-2018-100168. 31036563

[ref66] Woodward, L. J., & Fergusson, D. M. (2001). Life course outcomes of young people with anxiety disorders in adolescence. Journal of the American Academy of Child and Adolescent Psychiatry, 40(9), 1086–1093. doi: 10.1097/00004583-200109000-00018. 11556633

[ref67] World Health Organisation (WHO) (2017). Depression and other common mental disorders global health estimates. Geneva, Switzerland: World Health Organisation (WHO). Retrieved from https://apps.who.int/iris/bitstream/handle/10665/254610/WHO-MSD-MER-2017.2-eng.pdf?sequence=1.

