# Supplementary material for: Device-measured sedentary behaviour and anxiety symptoms during adolescence: a 6-year prospective cohort study
Source: Psychol Med. 2020 Dec 18;52(14):2962–71. doi: 10.1017/S0033291720004948 (PMC9693656; doi:10.1017/S0033291720004948)
Supplement: Supplementary file 1 [file S0033291720004948sup001.docx]

**Supplementary materials**

**Figure s1. Flow chart of participants**

Participants who completed a CIS-R score at age 18

N = 4,257

Participants with accelerometer data for ≥3 days at age 16

N = 1,922

Participants with accelerometer data for ≥3 days at age 14

N = 3,574

Participants with accelerometer data for ≥3 days at age 12

N = 5,252

ALSPAC study population

N = 15,454 pregnancies

Children alive at 12 months

N = 14,901

*ALSPAC: Avon Longitudinal Study of Parents and Children.*

*NB, we presented this same figure in an earlier publication* (1)*.*

**Figure s2. A Directed Acyclic Graph (DAG) of associations between sedentary behaviour and anxiety**


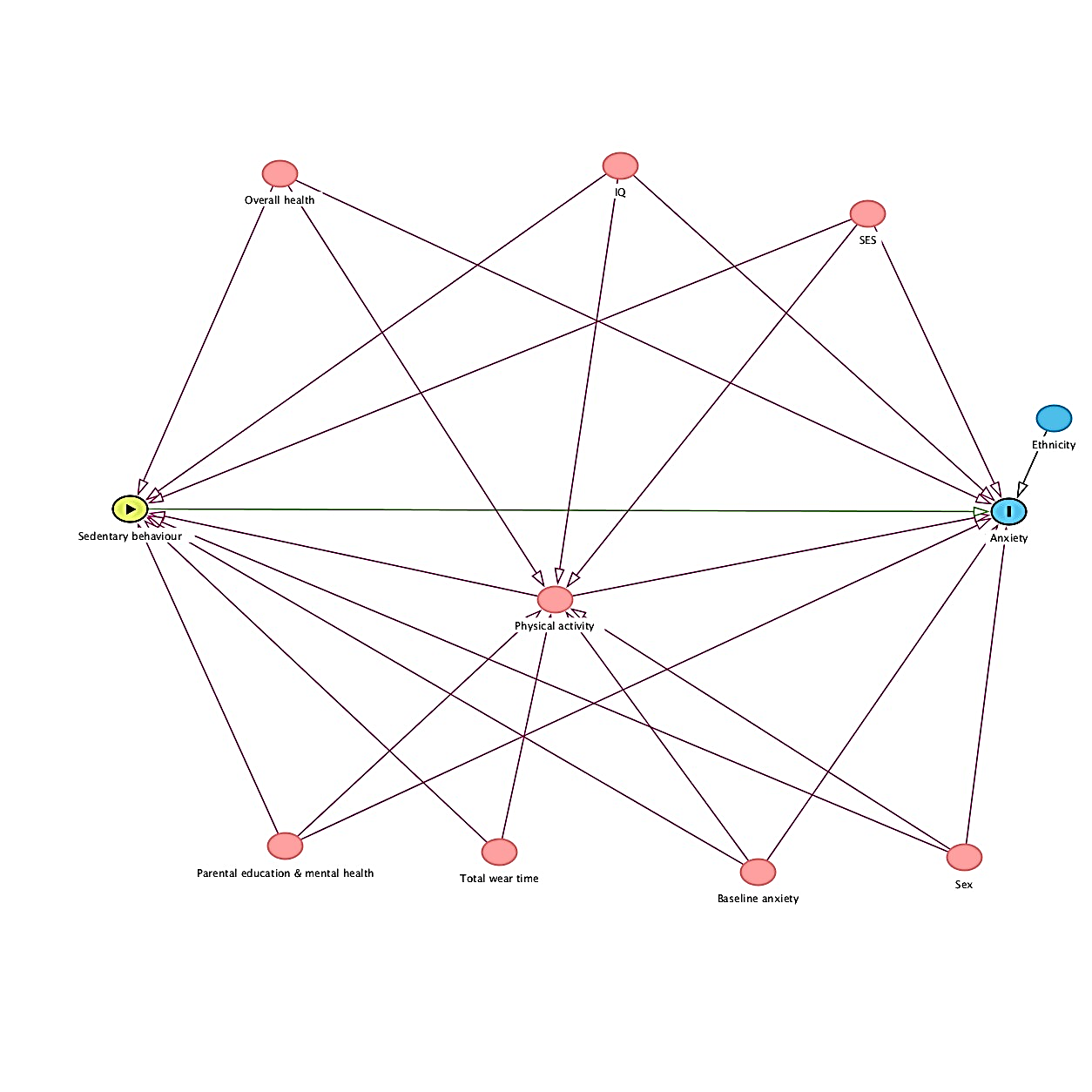


*NB, we constructed this DAG using the online resource:* [*http://www.dagitty.net/dags.html#*](http://www.dagitty.net/dags.html)*. Discussions between co-authors produced the causal assumptions underlying our analysis, which are graphically represented in this DAG. For simplicity, we have omitted arrows between covariates except for physical activity.*

**Figure s3. Distribution of anxiety scores (CIS-R)**

**Table s1. Baseline characteristics of included and excluded participants**

| Characteristics |  | Incidence or mean(SD)/total participants with available data (%) | | *P* |
| --- | --- | --- | --- | --- |
|  |  | **Included (n = 4,257)** | **Excluded (n = 10,664)** |  |
| Sex | Female | 2,390/4,257 (56.14) | 4,351/10,664 (40.80) | <0.001 |
| Ethnicity | Non-white | 173/4062 (4.26) | 429/7,912 (5.42) | 0.006 |
| Parental education | Higher (degree) | 1,207/4,257 (28.35) | 1,328/10,664 (12.45) | <0.001 |
| Maternal social class | Manual | 553/3626 (15.25) | 1,435/6,354 (22.58) | <0.001 |
| Parental psychiatric history | Severe depression or schizophrenia | 422/4257 (9.91) | 1,117/10,664 (10.47) | 0.065 |
| BMI | Overweight or obese | 270/3671 (7.35) | 263/2602 (10.11) | <0.001 |
| IQ | Mean (SD)/n | 107.50 (16)/3620 | 100.70 (16.3)/3,316 | <0.001 |
| Baseline anxiety | DAWBA | 64/3,584 (1.81) | 87/3,813 (2.28) | 0.156 |

*OR: Odds ratio; CIs: Confidence intervals; DAWBA = Development and Well-being Assessment; IQ = Intelligence quotient*

**Methods s1. Multiple imputation model**

We generated 30 datasets containing fully imputed data from all 4,257 participants in our sample using multiple imputations with chained equations (2). The chained equation method uses a separate condition distribution per imputed variable. Results from all 30 datasets are pooled into a single multiple imputation dataset. The dataset contain adjusted standard errors and account for the uncertainty of each prediction as multiple predictions are made for each value. The multiple imputation model contained the same variables as in a previous paper in the same cohort (1). We also included additional variables for measures of anxiety from the CIS-R and DAWBA and physical illness. We use predictive mean matching for non-normally distributed variables.

**Table s2. ISM in full cohort with imputed data**

| Age |  |  | Activity category | | | |
| --- | --- | --- | --- | --- | --- | --- |
|  | **Activity category** | | **Light** | | **MVPA** | |
|  |  |  | **%Δ** | **95% CI** | **%Δ** | **95% CI** |
| 12 | **Sedentary** | | -10.29 | -16.02, -4.17 | -25.90 | -43.50, -2.87 |
| 14 |  | | -9.78 | -16.71, -2.27 | 8.37 | -16.70, 2.27 |
| 16 |  | | -9.06 | -17.61, 0.36 | -10.40 | -32.53, 19.00 |

**Table s3. ISM with depressive symptoms as a confounding variable**

| Age |  |  | Activity category | | | |
| --- | --- | --- | --- | --- | --- | --- |
|  | **Activity category** | | **Light** | | **MVPA** | |
|  |  |  | **%Δ** | **95% CI** | **%Δ** | **95% CI** |
| 12 | **Sedentary** | | -15.81 | -22.36, -8.66 | -12.33 | -35.92, 19.99 |
| 14 |  | | -11.22 | -19.27, -2.37 | 5.70 | -22.1, 43.42 |
| 16 |  | | -9.58 | -20.07, 2.28 | -3.38 | -32.30, 36.17 |

**Table s4. ISM with smoking and alcohol use (age 16) as confounding variables**

| Age |  |  | Activity category | | | |
| --- | --- | --- | --- | --- | --- | --- |
|  | **Activity category** | | **Light** | | **MVPA** | |
|  |  |  | **%Δ** | **95% CI** | **%Δ** | **95% CI** |
| 12 | **Sedentary** | | -16.41 | -22.79, -9.49 | -7.62 | -32.42, 26.38 |
| 14 |  | | -12.17 | -20.11, -3.46 | -10.99 | -17.71, 49.53 |
| 16 |  | | -9.72 | -21.45, 3.77 | -4.50 | -34.92, 40.14 |

*NB smoking and alcohol are entered into the model at age 16.*

**Table s5. ISM with physical illness as a confounding variable**

| Age |  |  | Activity category | | | |
| --- | --- | --- | --- | --- | --- | --- |
|  | **Activity category** | | **Light** | | **MVPA** | |
|  |  |  | **%Δ** | **95% CI** | **%Δ** | **95% CI** |
| 12 | **Sedentary** | | -16.32 | -22.74, -9.38 | -7.52 | -32.40, 26.49 |
| 14 |  | | -11.71 | -19.72, -2.91 | 12.28 | -16.74, 51.41 |
| 16 |  | | -14.26 | -23.86, 3.44 | -3.35 | -30.95, 35.27 |

*NB physical illness was entered into all models as it was not possible to determine when the illness occurred.*

**Table s6. ISM excluding anyone with a possible anxiety disorder at baseline**

| Age |  |  | Activity category | | | |
| --- | --- | --- | --- | --- | --- | --- |
|  | **Activity category** | | **Light** | | **MVPA** | |
|  |  |  | **%Δ** | **95% CI** | **%Δ** | **95% CI** |
| 12 | **Sedentary** | | -16.02 | -22.47, -9.04 | -8.15 | -32.94, 25.82 |
| 14 |  | | -12.09 | -20.07, -3.31 | -12.60 | -16.55, 51.96 |
| 16 |  | | -14.65 | -24.18, -3.93 | -5.88 | -32.66, -31.55 |

**Table s7. ISM using linear regression**

| Age |  |  | Activity category | | | |
| --- | --- | --- | --- | --- | --- | --- |
|  | **Activity category** | | **Light** | | **MVPA** | |
|  |  |  | ***β*** | **95% CI** | ***β*** | **95% CI** |
| 12 | **Sedentary** | | -0.22 | -0.32, -0.12 | -0.09 | -0.48, 0.31 |
| 14 |  | | -0.16 | -0.28, -0.04 | 0.12 | -0.26, 0.49 |
| 16 |  | | -0.17 | -0.31, -0.03 | -0.08 | -0.49, 0.32 |

**Table s8. ISM with BMI as a confounding variable**

| Age |  |  | Activity category | | | |
| --- | --- | --- | --- | --- | --- | --- |
|  | **Activity category** | | **Light** | | **MVPA** | |
|  |  |  | **%Δ** | **95% CI** | **%Δ** | **95% CI** |
| 12 | **Sedentary** | | -15.71 | -22.32, -8.454 | -10.73 | -35.17, 22.92 |
| 14 |  | | -12.06 | -20.01, -3.32 | -10.83 | 17.73, 49.29 |
| 16 |  | | -14.97 | -24.51, -4.23 | -4.45 | 32.60, -33.48 |
